# A child with hyperferritinemia: Case report

**DOI:** 10.1186/1824-7288-37-20

**Published:** 2011-05-12

**Authors:** Melania Serra, Filomena Longo, Antonella Roetto, Alessandro Sandri, Antonio Piga

**Affiliations:** 1 Division of Pediatrics and Thalassemia Center, Department of Clinical and Biological Sciences, University of Torino, S. Luigi Gonzaga Hospital, Regione Gonzole 10, Orbassano (TO), 10043, Italy; 2 Molecular Medicine and Oncology Laboratory, Department of Clinical and Biological Sciences, University of Torino, S. Luigi Gonzaga Hospital, Regione Gonzole 10, Orbassano (TO), 10043, Italy

## Abstract

Hereditary hyperferritinemia cataract syndrome (HHCS) is a rare condition caused by mutations in the gene coding for the light chain of ferritin; it does not lead to iron overload, but it is associated with the risk of developing a bilateral nuclear cataract also in childhood. On the contrary, a raise of serum ferritin levels is a common finding in pediatrics. We describe here a case of HHCS that offers some interesting clues for the daily practice. Our patient is a 6 year old Italian boy who came to our attention after some time of diagnostic uncertainties because of persistently high levels of ferritin with no apparent cause. We were guided to the suspect of this syndrome by the family history (5 members with various degrees of cataract developed in first infancy). High levels of serum ferritin and specific genetic testing (mutation A37C) confirmed the diagnosis. This case underlines the need of considering rare genetic syndromes, including hereditary hyperferritinemia cataract syndrome, in the differential diagnosis of raised serum ferritin in children and the importance of paying attention to family history in considering a patient with isolated raised levels of serum ferritin.

## Background

The finding of high levels of serum ferritin in a child is not unusual; as inflammatory marker, ferritin can be above normal limits even for minor problems such us upper airway infections, with no relation with iron disorders. In the common practice, only the persistence of high ferritin levels is usually honored by further investigations. Causes of acquired hyperferritinemia include chronic inflammatory conditions (i.e. anemia of chronic diseases, with shift of iron from circulation to storage sites) [1], autoimmune diseases, malignacies, hemolysis, myolisis and liver diseases [2]. Raised ferritin levels can also be caused by genetic conditions: the best known of them is genetic hemochromatosis, which is an hereditary disorder characterized by an increase in iron absorption and deposition in tissues like heart and liver, with variable severity and age of onset according to the type of mutation involved. Rare genetic causes of raised serum ferritin levels include hereditary hyperferritinemia cataract syndrome (HHCS), an autosomal dominant condition caused by mutation in the ferritin light chain gene, specifically in the iron-responsive element (*IRE*) in the 5-prime noncoding region; as a result, the inhibitory function of *IRE *may not act and L-ferritin molecules are abnormally produced [3]. This extremely rare syndrome is characterized by high serum ferritin levels without evidence of iron overload and no relevant consequence except visual impairment caused by cataract. In this report we describe a new case of this syndrome in an Italian family, whose diagnosis took place from the finding of high ferritin levels in an otherwise healthy 6 year old child.

## Case presentation

Our proband is an Italian boy born in 2003, who, at the age of 3, came to the attention of a pediatrician because of lack of appetite and was prescribed with general blood tests, which were normal except for serum ferritin (600 μg/L, normal values 15-120); this finding was not considered relevant. At the age of 6, after the advice from a second pediatrician, serum ferritin was retested and found abnormally high (1039 μg/L), with serum iron of 198 μg/dL (normal values 65-160) and serum transferrin of 243 mg/dL (normal values 200-360). The high values of ferritin were confirmed after 5 (1126 μg/L) and 6 months (1247 μg/L); serum transferrin and serum iron levels resulted repeatedly normal, with no anemia and normal hematological parameters. The patient's medical history documented a premature birth (31 weeks of gestational age) with subsequent respiratory distress disease and neonatal peritonitis; the following physical and cognitive development was good. Further investigations included liver function tests, thyroid function screening tests and abdominal ultrasound scan, which resulted normal. Genetic hemochromatosis was investigated only for the HFE gene, with the finding of heterozygosis of the mutation H36D; the mother was negative for HFE mutations and the father had H63D/C282Y heterozygosis. At this point, the family was referred to our Center for a further evaluation. We confirmed in the father the HFE related genetic hemochromatosis, with a pure expression (at the SQUID magnetic biosusceptometry, liver iron concentration of 7 mg Fe/g liver dry weight at 56 years of age). The child's physical evaluation was normal; growth parameters were found to be within the normal range (height at 50° percentile for age; weight at 25° percentile for age). Hematological tests performed during the consultation showed a normal blood test (hemoglobin concentration 13,2 g/dL) with mild reticulocytosis (80,8 × 1000/μL). Iron parameters were the following:

i. serum iron 96 μg/dL (normal values: 30-145)

ii. serum transferrin 222 mg/dL (normal values 200-360)

iii. transferrin saturation 34% (normal values 20-25)

iv. TIBC (total iron binding capacity) 277 mg/dL (normal values 300-470)

v. serum ferritin 1407 μg/L (normal values 20-300)

All the screening tests, including inflammatory markers, were normal. The SQUID demonstrated the absence of iron accumulation (LIC 2,2 mg Fe/g liver dry weight) and the transient elastometry by Fibroscan excluded hepatic fibrosis (liver stiffness 3,5 kPa, normal values < 5 kPa).

With these results, we restricted the differential diagnosis to genetic forms of hyperferritinemia without iron overload, starting from HHCS. The family history and further investigations took us easily to confirm our suspect: the mother and 4 other maternal relatives (grandmother, uncle, great uncle and aunt) had high serum ferritin levels and a congenital form of bilateral cataract requiring surgical intervention in two of them (46 years of age for the uncle, 30 years for the aunt); the proband's mother cannot undergo the operation because of a coexisting retinopathy. The subsequent analysis of the promoter of the L ferritin gene showed the mutation A37C in heterozygosis in the child and in his mother, giving the molecular background to the diagnosis. Our proband did not show any visual deficit at the time of the consultation nor any lenticular opacity at the ophthalmological evaluation, and is now on regular follow up in order to detect early signs of cataract.

## Discussion

This case enlightens the fact that the diagnosis of a rare syndrome can be suspected on the basis of the gathering of the family history of a child.

Persistently high serum ferritin levels in a child include very different causes, which can be acquired or genetically determined.

Hereditary hyperferritinemia-cataract syndrome (HHCS) is a rare genetic syndrome (80 families detected worldwide until September 2009 [4]) which was first described, almost simultaneously in 1995, by Girelli and colleagues in Verona, Italy [5,6], and by Bonneau and collegues in Poitier, France [7,8]; it is caused by a mutation of the gene coding for ferritin L chain on chromosome 19 (19q13.1), involving the *IRE *sequence. In the normal subject, the interaction between *IRE *and the cytoplasmic protein IRP causes a down-regulation of the synthesis of ferritin and this happens in relation to the iron status in the organism (in case of high iron levels in the organism, the *IRE*-IRP interaction is enhanced). Mutations of *IRE *involved in hyperferritinemia-cataract syndrome cause the inhibition of the *IRE*-IRP interaction, thus determining an accumulation of ferritin L chains, not related with the iron status [3]. The A37C mutation we found in our family had been previously reported by Cremonesi in Italy [4].

The pathogenesis of this syndrome explains the absence of iron overload. In the past, the evaluation of iron accumulation required invasive methods such as liver biopsy; at present, we tend to use non invasive techniques such as magnetic resonance imaging [9] or SQUID (superconducting quantum interference device) biosusceptometry [10]. In our case, with non invasive SQUID we had the opportunity to exclude the presence of liver iron overload with no discomfort for the child.

Other genetic causes of high ferritin levels in a child include juvenile hemochromatosis, an autosomal recessive disorder caused by the mutation of *HJV *(*HFE2*) gene [11], or, more rarely, of *HAMP *(*HEPC*) gene [12]; in contrast with hyperferritinemia-cataract syndrome, it is characterized by an early-onset iron overload, causing liver cirrhosis, cardiomyopathy and endocrine complications in the second or third decade of life; this condition may cause increased levels of ferritin and transferrin saturation in children [13,14]. By contrast, the most common cause of genetic hyperferritinemia with iron overload is hereditary hemochromatosis, which is caused by the mutation of the *HFE *gene [15]; the difference with both hyperferritinemia-cataract syndrome and juvenile hemochromatosis is that high ferritin levels in hereditary hemochromatosis cannot be seen in pediatric age, because iron overload develops through adulthood. Other rare genetic causes of high ferritin levels with effective iron overload in pediatric age include: aceruloplasminemia, which is characterized by a lack of ceruloplasmin, anemia and the development of iron overload, with neurological symptoms related to the deposition of iron in the cerebral tissues [16]; transferrin receptor 2-related hemochromatosis, which can sometimes have its onset before adulthood [17]; atrasferrinemia and hypotransferrinemia, in which hemosiderosis is accompanied by hypochromic microcytic anemia [18].

In our case, it was the association of high ferritin levels and cataract in some of the child's relatives which oriented us towards the suspect of hereditary hyperferritinemia-cataract syndrome.

In fact, the only pathological finding in hyperferritinemia-cataract syndrome is a bilateral nuclear cataract, whose pathogenesis is not completely understood. It has been demonstrated that lens opacities are caused by L-ferritin molecules aggregates [19-21], causing small light-diffracting opacities. The mechanism causing this phenomenon is not fully understood. These ferritin aggregates probably accumulate in the lens because of a mRNA overexpression and a very slow protein turnover typical of this body compartment, and also because the lens is a non-vascular structure surrounded by a crown, so ferritin cannot leave it easily [19]. As the cataract is not always congenital, but develops through time [22-24], it can be absent in the pediatric patient; in this case, for instance, our patient did not show any visual impairment at the time of examination, and other observations in literature [25,26] confirm that visual complaints in this syndrome are usually noted in the second to fourth decade of life.

This case shows how important the family history is in helping the pediatrician to reach a correct diagnosis. This concept, applicable to a very wide range of pathologies, is particularly important when rare diseases are concerned; it may happen, as in the example reported above, that the child's relatives are not aware of their hereditary condition, so the clinician must ask very direct questions about the existence of specific health problems in the family. This observation is concordant with the need to spend quality time with the family, in order to achieve precious information that can be essential in directing subsequent investigations.

## Conclusions

This case is an example of the detection of a rare genetic disorder in an apparently healthy child. It also enabled us to describe a new case of hereditary hyperferritinemia-cataract syndrome. Our case underlines the fact that high levels of ferritin do not always correlate with iron overload and that a thorough collection of the family history is essential for directing the pediatrician's investigation towards the hyperferritinemia-cataract syndrome.

## Consent

Written informed consent was obtained from the patient's parents for publication of this case report. A copy of this written consent is available for review by the Editor-In-Chief of this journal.

## Competing interests

The authors declare that they have no competing interests.

## Authors' contributions

MS, FL, AS and AP collected information from the family, wrote and revised the case report; AP and FL lead the clinical part of the diagnostic process; AR supervised the molecular analysis; all Authors participated in drafting the manuscript, then read and approved its final version.
